# Sleep Behaviour in Sickle Cell Disease: A Systematic Review and Meta-Analysis

**DOI:** 10.3390/children12010021

**Published:** 2024-12-26

**Authors:** Melanie Koelbel, Fenella J. Kirkham

**Affiliations:** Developmental Neurosciences Unit, Biomedical Research Centre, UCL Great Ormond Street Institute of Child Health, London WC1N 1EH, UK; melanie.koelbel.15@ucl.ac.uk

**Keywords:** sickle cell disease, sleep behaviour disorders, polysomnography, actigraphy, sleep diary, total sleep time, sleep onset latency, systematic review, meta-analysis

## Abstract

**Background/Objectives:** There is a high prevalence of sleep behaviour disorders, as well as sleep disordered breathing (SDB), in individuals living with sickle cell disease (SCD). SDB has been systematically reviewed; therefore, this systematic review and meta-analysis focused on sleep behaviour. **Methods:** The comprehensive literature search, following PRISMA reporting guidelines, included all languages, conference proceedings and published theses from inception through February 2022. We identified 31 studies, with most of the research being conducted in North America, using polysomnography, actigraphy and questionnaires/diaries in paediatric SCD cohorts. **Results:** Total sleep time (TST) decreased, while sleep onset latency (SOL) increased with age. TST was higher on self-reported sleep diary measures and lower on polysomnography (PSG) and actigraphy assessments. SOL was lowest during PSG and highest in actigraphy. The discrepancy between sleep measures might be due to the overestimation of sleep behaviour by parents. In six studies, TST and SOL were compared between people living with SCD and healthy controls; in four, TST was longer in those living with SCD while it was shorter in two. Meta-analyses on the effect of TST and SOL were limited due to publication bias, with heterogeneity between the studies, in part related to measurement differences. No significant differences were found. **Conclusions:** The scarcity of case-control studies and significant heterogeneity in findings likely attributable to variations in sleep assessment methodologies. Gaps in the literature should be addressed.

## 1. Introduction

The healthy human red blood cell is shaped like a biconcave disc, making it easy to move smoothly around the human vascular system, important for the continuous transport of oxygen, essential for cellular respiration. The most common recessively inherited red blood cell disorder is sickle cell disease (SCD). Patients are anaemic because the life span of the sickled cells is shorter. SCD is identified as a neurodevelopmental disorder because of the multifaceted impact of genes, vascular health, and social and environmental factors on early brain development [1]. Deficient sleep quality and chronic hypoxia, hallmarks of sleep-disordered breathing such as obstructive sleep apnoea, are hypothesised to compromise oxygen delivery, elevating the risk for structural and functional abnormalities within the brain, potentially leading to impaired cognitive function in individuals living with SCD [2].

Impaired sleep quality can negatively affect cognitive function in the general population [3]. Given the high prevalence of cognitive difficulties among children and young adolescents living with SCD [2,4,5], it is crucial to investigate whether sleep disturbances may contribute to these challenges in this vulnerable population. However, knowledge gaps regarding the precise impact and relationship of sleep behaviour in individuals living with SCD on overall health and cerebral function persist. Sleep behaviour and sleep disorders might play an important role in the development of these neurocognitive impairments, but the available data have not previously been systematically reviewed.

### 1.1. Prevalence of Sleep Disorders in Sickle Cell Disease

Half of children living with SCD were reported to show sleep onset insomnia (i.e., inability to fall asleep at bedtime and to stay asleep at night) and 21% of them experienced long-term insomnia [6], compared to 15–19% in the general paediatric population [7,8]. A recent review has shown that pain crisis and disease severity can have a significant impact on the quality of life in adults living with SCD [9], especially since their experienced pain can promote insomnia [10]. Nearly three quarters (71%) of adults living with SCD reported sleep disturbances and 21% showed signs of depression [11], which were correlated and more common in those with frequent pain. Children and adolescents living with SCD (8–18 years) who experienced more pain also showed lower sleep efficiency (measured with actigraphy) [12]. However, questions on the relationship between sleep-disordered breathing, pain severity and exacerbation of SCD remain [13,14].

Individuals living with SCD are at increased risk of sleep-disordered breathing since they often have hyperplasia of lymphoid tissue such as adenoids and tonsils, which is one of the main reasons for upper airway obstruction [15,16]. Sleep-disordered breathing occurs in 36–69% of people [17,18,19], while around half of the children living with sickle cell disease snore regularly [6]. Habitual snoring in these children was shown to be a risk factor for obstructive sleep apnoea [20]. Rosen et al. identified a high prevalence of sleep-disordered breathing in their study of children living with SCD in the UK and USA, with 34% of habitual snorers exhibiting an obstructive apnea–hypopnea index greater than ≥1. A recent review investigating obstructive sleep apnoea prevalence identified a high occurrence in both children and adults living with SCD [21]. The findings indicated that 51% of children and 43% of adults with SCD experienced obstructive sleep apnoea, as defined by an apnea–hypopnea index (AHI) ≥ 5.

Sleep quality is disturbed by multiple obstructive sleep apnoea events at night [22,23,24]. Research in the general population has linked obstructive sleep apnoea to various pathologies such as obesity [25], cognitive difficulties [26,27,28], daytime sleepiness [29], inflammation [30,31], oxidative stress and endothelial signal alterations [32,33].

The impact of sleep-disordered breathing on the pathogenesis of SCD is still not well understood. It is known that the increased risk of sleep-disordered breathing in these patients may result in a greater risk of nocturnal hypoxaemia [17,34,35] and hypercapnia [36]. Samuels et al. identified that 16% of children and adolescents living with SCD experienced intermittent hypoxaemia, which is likely to increase not only the polymerisation of the sickle red cells, but also hypertension and hence, the risk of vascular occlusion. Lower oxygen saturation at night associated with sleep-disordered breathing [20] was associated with a higher rate of central nervous system events [34] and painful crises [37] in the East London cohort. Functional outcomes may also be at risk because of a higher prevalence of silent infarction and microvascular brain changes, as observed in the general adult population [21,38,39,40].

### 1.2. Sleep Assessments

Multiple outcome measures contribute to the definition of sleep quality, including sleep quantity (total duration of sleep), sleep onset latency (time required to fall asleep), and wakefulness after sleep onset. A variety of sleep assessment techniques have been established to quantify sleep quality and sleep patterns, employing either subjective self- or parent-reported questionnaires [41], objective in-lab polysomnography [42] or at-home actigraphy [43] evaluations.

#### 1.2.1. Questionnaires

Sleep diaries are a cost-effective method for collecting self-reported aspects of sleep behaviour, sleep disruption and habits (e.g., total sleep time, wake time, nighttime awakenings). A few questionnaires have been developed to document sleep behaviour and sleep pathology (e.g., restless legs and sleep-disordered breathing). A systematic review in the general paediatric population found that the Paediatric Sleep Questionnaire (PSQ) [44] was widely used and sensitive to polysomnography measures, such as detecting sleep-disordered breathing symptoms [45]. This 22-item questionnaire asks about sleep behaviour (e.g., snoring frequency, difficulty breathing and daytime sleepiness). The Epworth Sleepiness Scale (ESS) [46] is an 8-item questionnaire that assesses the chances of dozing off or falling asleep while engaged in eight different activities (e.g., sitting and reading). Variations of the Children’s Sleep Habits Questionnaire (CSHQ) questionnaire developed by Judith Owens are also widely used [47,48]. The original CSHQ is a 45-item caregiver-rated questionnaire, which assesses paediatric sleep difficulties during the past week on a 3-point Likert scale. The sum of all CSHQ scored questions calculates a sleep disturbances score, with a range of 33 to 99 (>41 is suggestive of a paediatric sleep disorder). However, the validity of these sleep diaries and questionnaires relies on participant compliance, as it necessitates the accurate and consistent recording of sleep behaviour [49].

#### 1.2.2. Polysomnography

Although sleep diaries represent the most prevalent subjective method for sleep assessment, polysomnography stands as the gold standard in this domain. It enables the comprehensive objective measurement of distinct sleep stages (NREM and REM) and the clinical assessment for sleep disorders (e.g., sleep-disordered breathing and limb movement) [50]. It usually involves an in-hospital or at home sleep study for at least one full night [51]. Different physiological measurements, such as heartrate, breathing pattern and electrophysiological activity of the brain, are taken. For example, a nasal cannula can help to identify the complete or partial cessation of airflow through the upper airways. In children, this is typically secondary to mechanical obstruction by enlarged tonsils and adenoids or abnormal airway anatomy [16]. Different medical conditions can be observed during a polysomnography recording, such as apnoea, a temporary cessation of breathing, and hypopnea, a reduction in airflow. These obstructions cut off the oxygen supply to vital organs and restrict the removal of toxic carbon dioxide. This generally only occurs for a few seconds before the individual wakes up (arouses) slightly and often unknowingly. Different indices are used to categorise different breathing obstructions. In paediatrics, it is common to use the obstructive apnoea and hypopnoea index (e.g., OAHI ≥ 1) or apnoea and hypopnoea index (e.g., AHI ≥ 1) [52]. Oxygen saturation is measured using pulse oximetry during PSG, which may also detect central apnoea and hypopnoea where there is no evidence of obstruction.

However, there are limitations for polysomnography in terms of cost-effectiveness, time investment, and accessibility, hindering its application in obtaining broad and immediate, as well as long-term, insights into individual sleep behaviours. Research has emphasised the importance of considering night-to-night variability in patients, which can influence obtained sleep measures (e.g., apnoea–hypopnoea index, used to measure severity of obstructive sleep apnea) [53]. In comparison to assessments conducted within a hospital setting, at-home polysomnography offers a potentially more ecologically valid approach. Research suggests a successful implementation of this method in paediatric populations, with studies reporting a high rate of successful administration (87%) [54].

#### 1.2.3. Actigraphy

Actigraphy offers a low-burden, cost-effective and ecologically valid method for assessing sleep, circadian rhythms, and movement. This technique utilises a wearable accelerometer, typically in a watch-like format, worn on the non-dominant hand, to objectively record sleep behaviours over an extended period of time [55]. It can provide detailed information on day- and night-time movement. A potential limitation to actigraphy research is the lack of standardised algorithms [56]. Patterson et al. identified, through a systematic evaluation, that various actigraphy devices utilise different algorithms to quantify sleep and activity. This heterogeneity in algorithms can hinder the comparability of sleep data across studies employing different actigraphy devices. However, polysomnography and actigraphy are established methods for acquiring information on sleep, which can even be applied in cases of children with neurodevelopmental disorders [57,58].

### 1.3. Aims

To initiate the investigation of this multifaceted issue, it is crucial to comprehend an understanding of sleep patterns, sleep behaviour and potential disorders in this vulnerable population of children living with SCD, given the high prevalence of co-existing sleep-disorders. To investigate the convergence of various sleep measures, the previous use of these measures was assessed in studies of people living with SCD. This is important to gain insights into the true sleep patterns of children and young adolescents living with SCD, thereby facilitating the formulation of robust conclusions and the development of targeted interventions. To achieve this, a systematic review of the literature was conducted, and a meta-analysis of case-control studies was attempted. This approach allowed for critical evaluations of the current literature on sleep characteristics in individuals living with SCD.

### 1.4. Hypotheses

(1) Sleep behaviour (i.e., total sleep time) and sleep disorders (i.e., sleep-disordered breathing) are at least as common in individuals living with SCD as in the general population.
(2) Different sleep assessments show similar prevalences of sleep behaviour and sleep disorder.
(3) Individuals living with SCD experience significantly different sleep behaviours to healthy controls.

## 2. Materials and Methods

The current systematic review and meta-analysis was conducted following the Preferred Reporting Items for Systematic Reviews and Meta-Analyses (PRISMA) statement [59]. The review was not registered with Prospero.

### 2.1. Eligible Studies

Studies were included if they reported information on all of the following: (1) patient demographics, (2) sleep behaviour (i.e., total sleep time, sleep onset latency, wake after sleep onset), (3) sleep disorder (i.e., sleep-disordered breathing: apnoea–hypopnoea index and/or obstructive apnoea–hypopnoea index), (4) clinical measure of oxygen saturation at night, and (5) assessment method: polysomnography and/or actigraphy and/or sleep diary/ sleep questionnaire. Articles were excluded if they did not provide the required information for individuals living with SCD and/or healthy controls.

### 2.2. Search Strategy

A comprehensive literature search was performed to find appropriate articles. Searched databases included Cochrane Library, Ebsco, Embase, Google Scholar, Medline, Psychextra, PsycINFO, PubMed, ProQuest ResearchGate, Scopus, Web of Science, Opengrey and Zetoc. Manual searches looked at conference proceedings, published theses and references of included articles to identify eligible publications. The search included studies that assessed sleep behaviour in individuals living with SCD from inception through February 2022, with no date or language restriction. Prospero was searched to identify similar or identical systematic reviews currently in preparation, but no current review looking at sleep in individuals living with SCD was found. The search strategy used keywords including: “sickle cell”, “sleep”, “total sleep time”, “sleep apnoea/apnea”, ‘‘polysomnography”, “actigraphy”, “diary”, “sleep duration” and “sleep quality”.

### 2.3. Study Selection and Data Extraction

**Figure 1:**
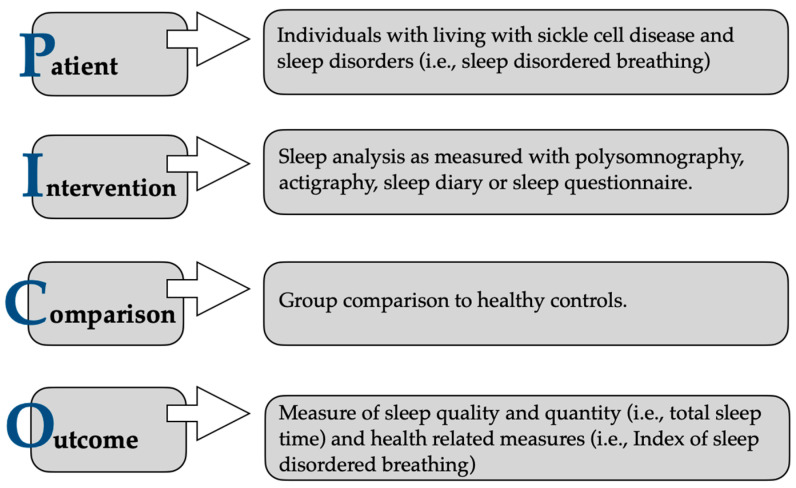
Inclusion criteria for adequate search guidance.

Study selection was based on title, abstract and final screening of full-text articles. Study selection was not limited by design, so randomised controlled trials, cohort studies and case-control studies were eligible. However, reviews and case studies were excluded from consideration. All potential studies were downloaded and catalogued using Mendeley reference manager and Excel. Duplicates were excluded, and after screening the abstracts of the remaining studies, a full-text screening of studies reporting sleep quality was performed and data were extracted in Excel and SPSS. Figure 1 displays the main inclusion criteria for the systematic review using the PICO-criteria. Data were analysed by 2 reviewers. The key information included the following: (1) study characteristics (year, country, assessment method), (2) patient characteristics (number, gender, age, genotype), (3) clinical characteristics (haemoglobin, oxygen saturation), (4) sleep characteristics (total sleep time, sleep onset latency, wake after sleep onset, sleep-disordered breathing). Articles were excluded if they did not provide the information required by the inclusion criteria.

### 2.4. Data Evaluation

The quality of all included studies was assessed using the Critical Appraisal Checklists (CASP-2024) (https://casp-uk.net/casp-tools-checklists/ accessed 24 October 2024). Two reviewers evaluated the methodological quality of the included studies using 12 criteria: (1) clarity of the research question, (2) adequacy of participant recruitment, (3) validity of exposure measurement, (4) validity of outcome measurement, (5) consideration and control of confounding factors, (6) appropriateness of follow-up, (7) clarity of results, (8) precision of results, (9) credibility of results, (10) applicability of results, (11) generalizability of results and (12) implications of the study. Each paper was assigned a quality rating of low (≤8), moderate (≤10), or high (≤12) based on a 12-point quality assessment checklist. Disagreements were resolved by consensus. The results are presented in Supplementary Materials Table S1. Overall, most of the studies showed a low (*n* = 13) methodological quality. Most studies lacked precision in their results (*n* = 14), failed to account for confounding factors (*n* = 11), and provided limited implications of their findings (*n* = 10). Only 2/6 case-control studies were identified to be of high quality. Despite the limitations of some studies, 9 of the cross-sectional and cohort studies were of high quality, and 7 were of moderate quality.

For the evaluation of sleep behaviour (e.g., total sleep time) mean(s) (M) ± standard deviation(s) (SD) of outcome measures were selected. In the case of missing or incomplete data, authors were contacted or the mean ± standard deviation were calculated from available data. Meta-analysis was conducted in SPSS version 28 for the subgroup comparison of the methodology used between individuals living with SCD and healthy controls (*n* = 6 studies) or for total sleep time and sleep onset latency (*n* = 5 studies). Random-effects models were used to account for the heterogeneity between the studies. Effect sizes for mean differences and 95% confidence intervals (CI) were reported. Forest plots were generated.

A pictorial representation was created to estimate a reference for typical sleep behaviour in individuals living with SCD based on 3 different sleep assessment methods (polysomnography, actigraphy and sleep diary), according to the mean and standard deviation of collected sleep data. In the pictorial representation, the sleep recommendation, based on different age groups, by previously published research by the National Sleep Foundation [60,61], was used as a reference point, and are the same and close to the recommendations by the American Academy of Sleep Medicine. They recommend that the following hours of sleep are necessary for optimal health: (1) children of 3 to 5 years—10 to 13 h; (2) children of 6 to 12 years—9 to 12 h; (3) teenagers of 13 to 18 years—8 to 10 h [62] and (4) adults—7 or more hours [63].

## 3. Results

**Figure 2:**
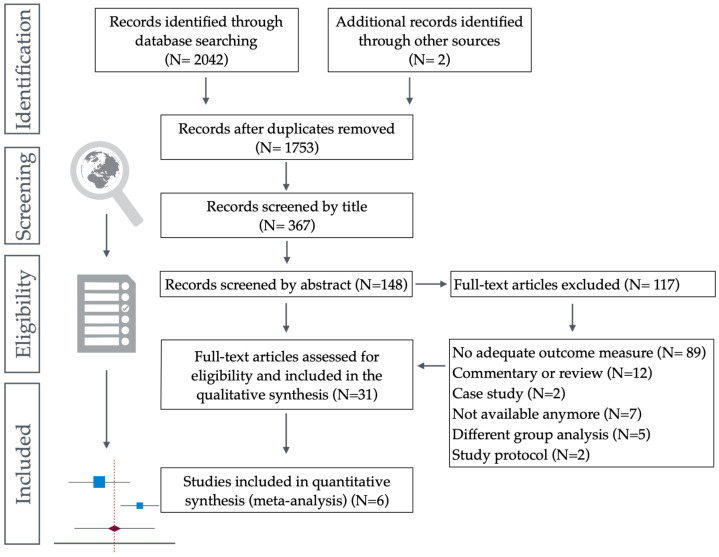
Flowchart for selection of studies for systematic review according to PRISMA guidelines.

The search identified 2044 records from the selected databases. Thirty-one articles were included in this review. Some articles provided sufficient data to allow for meta-analysis. A detailed selection is presented in Figure 2.

### 3.1. Study Population

The overall population characteristics are listed in Table 1. Sleep characteristics based on polysomnography (*n* = 20), actigraphy (*n* = 5) and sleep diaries and self-report (*n* = 6) are summarised in Table 2. Table 3 presents data on children and adults living with SCD (categorised by their sleep-disordered breathing group), differentiating these groups using various measures, including the apnoea–hypopnoea index (AHI) and the obstructive apnoea–hypopnoea index (OAHI).

The majority of studies looked at individuals living with SCD in North America (nUSA only = 17; nCanada = 1) (Table 1). Smaller groups looked at individuals living with SCD in Europe (nPortugal = 4; nUK only = 2), South America (nBrazil =2), Asia (nSaudi Arabia = 2), and Africa (nCameroon = 1). Two studies examined populations from the UK and USA (see Table 1). From the data that were available, the mean age ranged from 4.8 to 38.5 years, with 43% of the participants being male. Children living with SCD below the age of 18 years were included in 26 studies, while 4 studies looked at young adults living with SCD (Table 1). One paediatric study did not mention the age of the population [64]. A variety of genotypes were reported (nHbSS = 1179; nHbSC = 209; nHbSß = 88, nOther = 8), with a haemoglobin range of 7.86–10.6 g/dL (*n* = 11 studies) (Table 1).

An analysis of all of the included studies revealed a spectrum of sleep variables measured in individuals living with SCD. The following mean sleep variable ranges were identified for (1) total sleep time: 417–622.8 min, (2) sleep onset latency: 7.27–75 min, (3) mean overnight oxygen saturation (SpO_2_): 93–98%, (4) apnoea–hypopnoea index: 0–17 events/hour, (5) obstructive apnoea–hypopnoea index: 0–18.5 events/hour and (6) wake after sleep onset: 31.4–59.1 min (Table 2).

Among individuals living with SCD considered to have sleep-disordered breathing by various definitions, the mean age ranged from 7.6 to 41 years (Table 2). This subgroup comprised 48% males and included a variety of genotypes (nHbSS = 88; nHbSC = 16; nHbSß = 7, nOther = 4) with a mean haemoglobin range of 7.6–9 g/dL. Individuals living with SCD considered to have sleep-disordered breathing demonstrated a wider range of total sleep time (332.75–463 min) compared to those without sleep-disordered breathing (323–444.5 min). Conversely, sleep onset latency was slightly shorter in the sleep-disordered breathing group (12–39.2 min) compared to the non-sleep-disordered breathing group (11.15–42.3 min). Finally, oxygen saturation levels were lower in the sleep-disordered breathing group (93–96%) compared to the non-sleep-disordered breathing group (94–98%). Most of the studies did not find a significant difference between the sleep-disordered breathing and non-sleep-disordered breathing groups. However, Alotaibi et al. (2018) found a significant difference between the groups for sleep onset latency and oxygen saturation.

### 3.2. Identification of Total Sleep Behaviour

**Figure 3:**
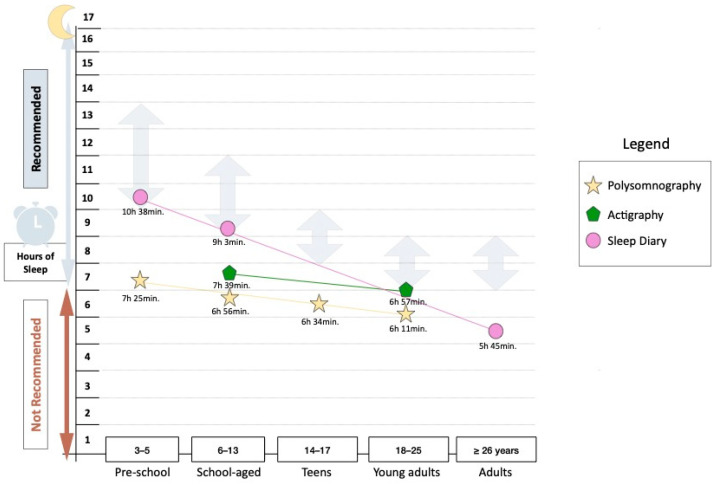
Total sleep time in sickle cell disease as observed with polysomnography. Note: Arrows show recommended sleep time as given by Ohayon (2017) [61].

Information on different sleep behaviours (e.g., total sleep time) was taken from studies that did not group individuals living with SCD according to their sleep-disordered breathing. Total sleep time, as measured by polysomnography, demonstrated a lower range (340–473 min) compared to both actigraphy (417–481 min) and sleep diary measures (420–623 min) (Figure 3).

There was a large mean difference in total sleep time (MΔTST) as measured with polysomnography in pre-school (MΔTST = 3 h and 13 min) and school-aged children (MΔTST = 2 h and 7 min) as compared to sleep diary measures. Smaller mean differences were observed between polysomnography vs. actigraphy (MΔTST = 43 min) and actigraphy vs. sleep diary measures (MΔTST = 1 h and 24 min) in school-aged children and polysomnography vs. actigraphy (MΔTST = 46 min) in young adults. Data collected from sleep diaries indicate that TST in children living with SCD is close to the recommended sleep time, but not for TST as measured with polysomnography and actigraphy. Unfortunately, there were not enough data available to compare TST measures in teenagers and adults.

### 3.3. Identification of Total Sleep Onset Latency

**Figure 4:**
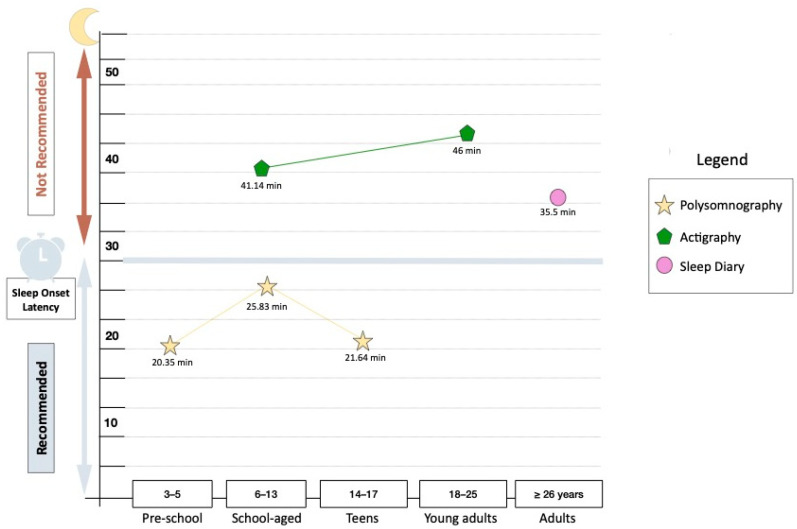
Sleep onset latency in sickle cell disease as observed with polysomnography, actigraphy and sleep diary measures. Note. Sleep onset latency below 30 min is an indication of a good sleep quality, as per the recommended sleep time given by Ohayon (2017) [61].

An analysis of sleep onset latency (SOL) across studies revealed the lowest range when measured using polysomnography (PSG) (12.7–53.9 min), followed by sleep diary measures (35.5 min) and actigraphy (7.27–75 min) (Figure 4). There was a large mean difference in SOL (MΔSOL) as measured with polysomnography and actigraphy (MΔSOL = 15.31 min) for school-aged children. There were insufficient data available to compare all measures in each age group. However, it was observed that SOL appeared to be lower when measured with polysomnography, compared to actigraphy and sleep diary measures.

### 3.4. Identification of Wake After Sleep Onset

Wake after sleep onset, a measure of sleep continuity, was only observed in studies that used polysomnography (range: 31.4–59.1 min). A higher than recommended wake after sleep onset was observed for pre-school children (M = 31.4 min) and school-aged children (M = 45.67 min), which was above the recommended time of 0–20 min.

### 3.5. Meta-Analysis

The aim was to investigate whether there were significant differences in total sleep time and sleep onset latency, as measured with the different sleep assessment methods, between individuals living with SCD and healthy controls. Data were available from six studies (Table 4). Table 4 shows all case-control studies included in the meta-analysis with the results between groups = for total sleep time and sleep onset latency displayed. A random effect model was chosen, since it can be assumed that the data are heterogenous in nature.

#### 3.5.1. Total Sleep Time

The first forest plot displays the effect of total sleep time, measured with polysomnography (Supplementary Materials Figure S1). The mean effect size estimate was −0.12 (95% CI: −0.59, 0.35) and statistically non-significant (*p* = 0.62). For heterogeneity, Q-statistics were examined. The Q-statistic (Q = 2.68, df = 1, *p* = 0.10) was found to be non-significant, indicating no heterogeneity between studies.

The second forest plot displays the effect of total sleep time, measured with actigraphy (Supplementary Materials Figure S2). The mean effect size estimate was −1.59 (95% CI: −5.32, 2.15) and statistically non-significant (*p* = 0.40). The Q-statistic (Q = 27.26, df = 1, *p* < 0.001) was found to be significant, indicating heterogeneity between the study conducted in Cameroon (Hedges’s g = −3.54, *p* < 0.001) and the UK (Hedges’s g = 0.27, *p* = 0.38).

The third forest plot displays the effect of total sleep time, measured with sleep diary (Supplementary Materials Figure S3). The mean effect size estimate was 0.38 (95% CI: −0.19, 0.94) and statistically non-significant (*p* = 0.19). The Q-statistic (Q = 5.18, df = 1, *p* = 0.023) was found to be significant, indicating heterogeneity between both studies conducted in the USA in 2010 (Hedges’s g = 0.078, *p* = 0.68) and in the USA in 2018 (Hedges’s g = 0.65, *p* < 0.001).

#### 3.5.2. Sleep Onset Latency

The forest plot displays the effect of sleep onset latency, measured with actigraphy (Supplementary Materials Figure S4). The mean effect size estimate was −1.15 (95% CI: −4.15, 1.86) and statistically non-significant (*p* = 0.45). The Q-statistic (Q = 22.55, df = 1, *p* < 0.001) was found to be significant, indicating heterogeneity between the study conducted in Cameroon (Hedges’s g = −2.72, *p* < 0.001) and the UK (Hedges’s g = 0.34, *p* = 0.26).

## 4. Discussion

This review summarised the literature on sleep behaviour and its association with sleep-disordered breathing in individuals with SCD. Thirty-one studies were included in the review; most were cohort studies and only six case-control studies qualified for the meta-analysis. Most of the studies were conducted in children, using polysomnography to measure their sleep behaviour and to obtain a clinical understanding of their sleep-disordered breathing symptoms. It is well known that the prevalence of sleep-disordered breathing (36% to 69%) is high in this vulnerable population and already present at a very young age [17,18,19].

This research identified that total sleep time decreases, and sleep onset latency increases with age. Overall, both outcome measures varied depending on which sleep assessment was used. For example, the range of total sleep time reported was wider on self-reported sleep diary and questionnaire measures, while total sleep time on objective measures (i.e., polysomnography and actigraphy) was less. However, this was the other way around for sleep onset latency; thus, the time taken to fall asleep was lowest during polysomnography assessment and highest in actigraphy. The findings on the decline in total sleep time are in line with current research typically in developing populations [65,66], including those of similar ethnic background [67]. This also accounts for the observed increase in sleep onset latency, showing that 38% of 8–12-year-old children living with SCD experience sleep onset difficulties [68], while 21% of 2–18 year-olds experienced chronic insomnia [6]. In the general paediatric population, only 11% of 5–12 year-olds experience sleep onset insomnia [69]. The differences observed between sleep assessments can be explained with findings from the general population, which compared parent-reported sleep with either actigraphy or polysomnography [70]. Nelson et al. (2014) found that parents overestimated sleep duration, as measured by actigraphy, by an average of 24 min [70]. Another study found that parents overestimated their children’s total sleep time, sleep onset latency and sleep efficiency when compared to overnight at-home polysomnography [71]. It is also important to mention that the variability of actigraphy data may contribute to the variability observed in algorithms used [72]. Research evaluating the reliability of in-hospital polysomnography have highlighted the importance of considering night-to-night variability in sleep parameters, which can influence diagnostic outcomes [53]. To address this variability, the authors suggested employing multiple nights of polysomnography recordings. However, this approach presents a significant cost burden. Recent research found a 90% success rate for in-hospital polysomnography employing a psychological preparation in children (2–11 years) (e.g., pictures, familiarising the child with the equipment, token sheets and a doll) 30 min to 1 h before the sleep assessment [73]. This could possibility reduce the night-to-night variability by reducing the child’s anxiety (i.e., promoting feelings of comfort and familiarity).

The majority of case-control studies reported that children and adolescents living with SCD slept longer, on average, compared to the controls [74,75,76,77]. Questionnaire assessment revealed longer total sleep times compared to actigraphy and polysomnography. However, two studies in children living with SCD found shorter total sleep times compared to the controls [64,78]. Notably, the findings by Njamnshi et al. are based on an abstract publication with a limited sample size, potentially reflecting the challenges associated with recruiting vulnerable populations in Cameroon. The main research focus for Strauss et al. was not the assessment of sleep behaviour, but the assessment of upper airway lymphoid tissue in children living with SCD and experiencing obstructive sleep apnea syndrome. Their findings could represent a subgroup of children living with SCD based on background (country) and the presence of comorbid health conditions.

Children living with SCD seem to fall asleep quicker compared to the controls [64,76], while our research [75] found slightly longer sleep onset latency. The lack of case-control studies investigating sleep in individuals living with SCD is further compounded by substantial heterogeneity in the reporting of total sleep time and sleep onset latency. This methodological inconsistency, evident in studies employing both actigraphy and sleep diaries, hinders a quantitative synthesis of the existing evidence.

## 5. Conclusions

There is a high prevalence of sleep disorders and sleep disturbances in individuals living with SCD, which are measured with different sleep assessments. An age-related change in sleep pattern was observed (i.e., decrease in total sleep time and increase in sleep onset latency). However, the scarcity of case-control studies and significant heterogeneity in findings likely attributable to variations in sleep assessment methodologies.

## Figures and Tables

**Table 1:** Summary of participant and study information of included studies that look at sleep behaviour in sickle cell disease.

Author	Year	Country	Design	Method	*n*	Genotype	Male *%*	hb g/dL	Age Years
Valrie et al.	2006	USA	Cross-sectional	Sleep Diary	20	HbSS = 14	54	n.a.	10.1 ± 1.07
						HbSC = 5			
						HbSß = 1			
Souza and Viegas	2007	Brazil	Cross-sectional	PSG	50	HbSS	50	8.3 ± 1.3	13.9 ± 2.5
Valrie et al.	2007	USA	Cohort	Sleep Diary	21	HbSS = 17	45	n.a.	10.13 ± 1.15
						HbSC = 6			
						HbSß = 1			
Kaleyias et al.	2008	USA	Case-control	PSG	19	n.a.	68	n.a.	10.7
									(6.4–13.3) *
Ferreira et al.	2009	Portugal	Cross-sectional	PSG	17	HbSS = 16	47	7.8	6.83
								(7.05–8.8) *	(3.16–9.25) *
Rogers	2009	USA	Cohort	PSG	45	HbSS = 32	47	8.3 ± 1.3	9.50
						HbSC = 9 HbSß0 = 2		10.5 ± 0.23	
								n.a.	
Salles et al.	2009	Brazil	Cross-sectional	PSG	85	n.a.	See Table 3 (AHI grouped)		
Daniel et al.	2010	USA	Case-control	CSHQ	54	HbSS = 28	56	n.a.	6.56 ± 1.92
						HbSC = 22			
						HbSß+ = 1 HbSß0 = 2 HbSJ_Baltimore_ = 1			
Martins et al.	2010	Portugal	Cross-sectional	PSG	6	HbSS	n.a	n.a.	28 ± 12
Rogers et al.	2010	USA	Cross-sectional	PSG	41	HbSS = 41	48	8.4 ± 1.3	9.4 ± 4.6
					14	HbSC = 14	29	10.6 ± 0.8	9.9 ± 4.7
Rogers et al.	2011	USA	Cross-sectional	PSG	64	HbSS = 64	50	8.2 ± 1.2	8.4 ± 4.8
Mullin et al.	2012	USA/UK	Cohort	PSG	45	HbSS or HbSß0	49	n.a.	12.3 ± 4
Strauss et al.	2012	USA	Case-control	PSG	36	n.a.	56	n.a.	6.9 ± 4.3
Finch et al.	2013	USA	Cohort	PSG	13	HbSS = 8	53	8.8 ± 1.7	7 (2.1–16.3) *
						HbSC = 4			
						HbSß0 = 1			
Njamnshi et al.	2013	Cameroon	Cross-sectional	Actigraphy	13	n.a.	n.a	n.a.	Children
Katz et al.	2014	USA	Case-control	PSG	136	HbSS = 77.5%	51	14.2 ± 5.2	n.a
						HbSC = 15.5%			
						HbSß0 = 7%			
Rosen et al.	2014	USA / UK	Cross-sectional	PSG	243	HbSS = 95%	50	n.a.	10.6 ± 4.2
Loureiro et al.	2015	Portugal	Cross-sectional	PSG	54	Group A = 21	57	n.a.	5.2 ± 1.7
						Group B = 33	55	n.a.	12.2 ± 2.4
Mascarenhas et al.	2015	Portugal	Case-control	PSG	65	n.a.	53	n.a.	9.4 ± 4.6
Moscou-Jackson et al.	2015	USA	Cross-sectional	Sleep Diary	75	HbSS, HbSC	28	n.a.	38.5 ± 11.8
Narang et al.	2015	Canada	Cross-sectional	PSG	104 HU−	HbSS = 101	42	8.2 (6.6–12.3) *	10.35 (2.70–17.70) *
						HbSC = 2			
						HbSß = 1			
					37 HU+	HbSS = 35	43	9.3 (7.3–11.6) *	10.90 (2.40–17.60) *
						HbSC = 1			
						HbSß = 1			
Sharma et al.	2015	USA	Cross-sectional	PSG	32	n.a.	See Table 3 (AHI grouped)		
Al-Otaibi et al.	2017	Saudi Arabia	Cross-sectional	PSG	65	HbSS = 90.8%	49	8.60	8.1 ± 5.02
Downes et al.	2017	UK	Case-control	CSHQ	22	HbSS = 22%	n.a	n.a.	4.8 ± 0.94
Alotaibi et al.	2018	Saudi Arabia	Cross-sectional	PSG	70	HbSS = 56	56	8.2 (7.8–9) *	9 (6.5–11) *
						HbSC = 7			
						HbSß = 7			
Fisher et al.	2018	USA	Cohort	Actigraphy	30	HbSS = 77%	33	n.a.	13 ± 2.8
Katz et al.	2018	USA	Cohort	PSG	136	HbSS = 95	51	9.3 ± 1.6	9.2 ± 4.7
						HbSC = 20			
						HbSß+ = 8			
						HbSß0 = 11			
Valrie et al.	2018	USA	Case-control	Self-report	53	HbSS = 27	42	n.a.	14.72 ± 1.50
						HbSC = 20			
						HbSß+ = 3 HbSß0 = 3			
Valrie et al.	2019	USA	Cohort	Actigraphy	88	HbSS = 44	41	n.a.	11.66 ± 2.99
						HbSC = 27			
						HbSß+ = 12			
						HbSß0 = 3			
Valrie et al.	2020	USA	Cohort	Actigraphy	96	HbSS = 44 HbSC = 35	44	n.a.	11.47 ± 3.03
						HbSß+ = 10 HbSß0 = 4			
Kölbel et al.	2022	UK	Case-control	Actigraphy	27	HbSS	41	n.a.	19.33 ± 5.16

Note. AHI = apnea–hypopnea index; Hb = haemoglobin in grams per decilitre; HU = hydroxyurea; n.a. = not available; PSG = polysomnography. Values as mean ± SD except where values are indicated with * = median (range).

**Table 2:** Summary of sleep behaviour in sickle cell disease of included studies.

Author	Year	Method	TST	SOL	SDB	WASO	O_2_
			Minutes		Minutes		%
Souza and Viegas	2007	PSG	410 ± 64	18 ± 20	AHI 2 ± 3	n.a.	
Kaleyias et al.	2008	PSG	384 (359–429) *	44 (17–53) *	AHI 1 (0–10) *	27 (9–75) *	n.a.
Ferreira et al.	2009	PSG	474 (435–489) *	40 (26.75–49.75) *	AHI 0 (0–0.45) *	n.a.	
Rogers	2009	PSG	431.80 ± 79.2	44.97 ± 64.6	OAHI 6.95 ± 12.9	44.35 ± 47.2	95.16 ± 3.9
Martins et al.	2010	PSG	371 ± 85	n.a.			93.0 ± 3.8
Rogers et al.	2010	PSG	430.6 ± 81.2	53.9 ± 70.5	OAHI 6.2 ± 11.7	46.6 ± 50.6	95.2 ± 3.8
			445.6 ± 53.1	26.1 ± 18.5	OAHI 3.1 ± 2.1	43.8 ± 28.7	98.0 ± 0.8
Rogers et al.	2011	PSG	432.6 ± 64.7	29.6 ± 31.6	OAHI 1.7 ± 3.7	n.a.	
Mullin et al.	2012	PSG	473.4 [451.0, 495.8]	n.a.	AHI 1.2 [0, 35.2] *	n.a.	94.5 [93.6, 95.5]
Strauss et al.	2012	PSG	438 ± 72	n.a.	AHI 1.9 ± 4.7	n.a.	95.3 ± 2.9
Finch et al.	2013	PSG	399.1 ± 98.9	17.1 ± 15.3	AHI 6.3 ± 5.8	n.a.	
Katz et al.	2014	PSG	378.02 ± 58.42	21.64 ± 27.18	AHI 8.51 ± 7.00	n.a.	95.63 ± 2.98
Rosen et al.	2014	PSG	438.7 ± 68.2	20.5 *	OAHI 0 *	n.a.	96.4 *
Loureiro et al.	2015	PSG	444.9 ± 39.1	20.7 ± 14.3	AHI 3.4 ± 1.3	31.4 ± 19.8	95.2 ± 3
			419.9 ± 57.2	20 ± 14.2	AHI 3.5 ± 1.9	59.1 ± 49.2	94.2 ± 3
Mascarenhas et al.	2015	PSG	424.8 ± 52.7	21.1 ± 13.6	AHI 3.57 ± 1.8	n.a.	94.5 ± 3.07
Narang et al.	2015	PSG	402 (248–524) *	11.4 (0.2–107.8) *	OAHI 1.9 (0.0–66.5) *	n.a.	96.1 (86.4–99.7) *
			406 (312–491) *	12.3 (0.4–181.1) *	OAHI 0.9 (0.0–14.3) *	n.a.	98.4 (91.6–99.6) *
Al-Otaibi et al.	2017	PSG	372.38	12.7	OA 0.35 (10) *	n.a.	98 (8) *
Alotaibi et al.	2018	PSG	344 (295–378) *	14.2 (4.3–37.5) *	OAHI 1.8 (0.3–6.3) *	n.a.	97 (96–98) *
Katz et al.	2018	PSG	377.102 ± 58.9	21.6 ± 27.2	AHI 8.6 ± 7.00	n.a.	95.6 ± 3.00
Njamnshi et al.	2013	Actigraphy	422.66 ± 33.24	75 ± 15.35	n.a.		
Fisher et al.	2018	Actigraphy	456.49 ± 105.91	n.a.			
Valrie et al.	2019	Actigraphy	481.8 ± 55.8	7.27 ± 2.55	n.a.		
Valrie et al.	2020	Actigraphy	477 ± 58	n.a.			
Kölbel et al.	2022	Actigraphy	388 ± 66	48 ± 42	n.a.		
Valrie et al.	2006	Sleep Diary	9.14 ± 1.29	n.a.			
Valrie et al.	2007	Sleep Diary	8.8 ± 1.65	n.a.			
Daniel et al.	2010	CSHQ	9.97 ± 1.93	n.a.			
Moscou-Jackson et al.	2015	Sleep Diary	7.0 ± 2.2	35.5 ± 35.4	n.a.		
Downes et al.	2017	CSHQ	10.38 ± 1.4	n.a.			
Valrie et al.	2018	Self-report	8.21 ± 1.64	n.a.			

Note. AHI = apnea–hypopnea index; CSHQ = Children’s Sleep Habits Questionnaire; SDB = sleep-disordered breathing; SOL = sleep onset latency; TST = total sleep time; OA = obstructive apnea; OAHI = obstructive apnea–hypopnea index; O_2_ = oxygen; WASO = wake after sleep onset; n.a. = not available. All values in mean ± SD. Values presented as mean ± SD, except values with * = median (range) or [ ] = 95% (CI).

**Table 3:** Summary of included studies that defined sleep-disordered breathing in sickle cell disease using polysomnography.

Author	Year	Group Description			Male	Hb	Age	TST	SOL	SDB	O_2_	WASO
		*n*	Genotype	SDB Group	*%*	g/dL		Minutes	Minutes	Events/h	%	Minutes
Salles et al.	2009	76	n.a.	No AHI	59	7.9 ± 2	9 ± 3	368 ± 63	22 (8–45) *	AHI 0 (0–0) *	94 ± 4	n.a
		9		AHI > 1		7.6 ± 0.6	9 ± 4	332 ± 79	31 (18–50) *	AHI 1.3 (1.9–5.1) *	93 ± 3	n.a
Rogers	2009	12	HbSS = 8		67	8.63 ± 1.6	11 ± 4.3	463.9 ± 56.7	39.2 ± 32.2	OAHI 0.4 ± 0.3	95.7 ± 2.5	40.2 ± 48.2
			HbSC = 1	OAHI < 1								
			Other = 3									
		19	HbSS = 14		47	8.81 ± 1.5	8.4 ± 4.3	421.6 ± 74.2	42.3 ± 46.5	OAHI 2.5 ± 1.1	95.5 ± 3.4	41.7 ± 53.9
			HbSC = 4	OAHI > 1 < 5								
			Other = 1									
		14	HbSS = 10	OAHI ≥ 5	29	9.0 ± 1.6	7.6 ± 5.4	444.5 ± 38.2	28.4 ± 27.4	18.5 ± 18.7	94.4 ± 5.5	54.9 ± 37.6
			HbSC = 4									
Sharma et al.	2015	18	n.a.	No AHI	22	n.a.	38 [32–44]	341 [312–370]	12 [3.6–21]	AHI 1.6 [0.98–2.1]	n.a.	n.a.
		14		AHI > 5	43		41 [35–47]	323 [281–366]	25 [0.96–48]	AHI 17 [10–24]		
Alotaibi et al.	2018	32	HbSS = 29	No OAHI	66	8.7 (7.9–9.4) *	9 (6–12) *	341 (305–378)	15.3 (7.0–40.8) *	OAHI 0.4 (0.0–1.0) *	98 (97–99) *	
			HbSC = 2									n.a.
			HbSß = 1									
		38	HbSS = 27	OAHI > 2	48	8.0 (7.8–8.6) *	9 (7–10) *	344 (293–379) *	7.4 (1.5–28.3) *	OAHI 6.5 (4.3–12.9) *	96 (94–98) *	n.a.
			HbSC = 5									
			HbSß = 6									

Note. Hb = haemoglobin in grams per decilitre; n.a. = not available; TST = total sleep time; SOL = sleep onset latency; SDB = sleep-disordered breathing; AHI = apnea–hypopnea index; OAHI = obstructive apnea–hypopnea index. O_2_ = oxygen; WASO = wake after sleep onset. Values presented as mean ± SD, except values with * = median (range) or [ ] = 95% (CI).

**Table 4:** Summary of included case-control studies.

Author			Group Description				Total Sleep Time		*p*	Sleep Onset Latency		*p*
	SCD			Controls								
	*n*	Age	Male %	*n*	Age	Male %	SCD	Controls		SCD	Controls	
Daniel et al.	54	6.56 ± 1.92	56	52	6.71 ± 2.05	48	637± 153	625 ± 151	0.747	n.a.	n.a.	n.a.
Strauss et al.	36	6.9 ± 4.3	56	36	6.6 ± 3.4	56	438 ± 72	462 ± 48	>0.05	n.a.	n.a.	n.a.
Njamnshi et al.	13	Children	n.a.	13	Children	n.a.	422.66 ± 33.24	528.75 ± 24.10	0.0084	75 ± 15.35	113.41 ± 11.79	0.02
Mascarenhas et al.	65	9.4 ± 4.6	53	65	9.4 ± 4.6	53.8	424.8 ± 52.7	419.8 ± 50.6	0.581	21.1 ± 13.6	30 ± 4.9	0.024
Valrie et al.	53	14.72 ± 1.50	42	160	15.36 ± 1.49	43	501 ± 124	426 ± 111	0.01	n.a.	n.a.	n.a.
Kölbel et al.	27	19.33 ± 5.16	41	18	19.43 ± 3.99	27	388 ± 66	371 ± 54	0.01	48 ± 42	35 ± 27	0.32

Note. Age in years, SCD = sickle cell disease, n.a. = not available, all values in mean ± SD.

## Supplementary Materials

The following supporting information can be downloaded at: https://www.mdpi.com/article/10.3390/children12010021/s1, Table S1: Quality assessment table. Figure S1: Forest Plot for total sleep time measured by polysomnography. Figure S2: Forest Plot for total sleep time measured by actigraphy. Figure S3: Forest Plot for total sleep time measured by sleep diary. Figure S4: Forest Plot for sleep onset latency measured by actigraphy.

## References

[1] Schatz J., McClellan C.B. (2006). Sickle cell disease as a neurodevelopmental disorder. Ment. Retard. Dev. Disabil. Res. Rev..

[2] Stotesbury H., Kirkham F.J., Kölbel M., Balfour P., Clayden J.D., Sahota S., Sakaria S., Saunders D.E., Howard J., Kesse-Adu R. (2018). White matter integrity and processing speed in sickle cell anemia. Neurology.

[3] Cousins J.N., Sasmita K., Chee M.W.L. (2018). Memory encoding is impaired after multiple nights of partial sleep restriction. J. Sleep Res..

[4] Allick A., Rao E., Casella J.F., Slifer K., Cannon A., Jiang H., Chin E., Lance E. (2023). Global White Matter Changes and Associations with Cognition in Pediatric Sickle Cell Disease. Blood.

[5] Kawadler J., Clayden J.D., Clark C.A., Kirkham F.J. (2016). Intelligence quotient in paediatric sickle cell disease: A systematic review and meta-analysis. Dev. Med. Child Neurol..

[6] Hankins J.S., Verevkina N.I., Smeltzer M.P., Wu S., Aygun B., Clarke D.F. (2014). Assessment of sleep-related disorders in children with sickle cell disease. Hemoglobin.

[7] Calhoun S.L., Fernandez-Mendoza J., Vgontzas A.N., Liao D., Bixler E.O. (2014). Prevalence of Insomnia Symptoms in a General Population Sample of Young Children and Preadolescents: Gender Effects Susan. Sleep Med..

[8] Cao X.L., Wang S.B., Zhong B.L., Zhang L., Ungvari G.S., Ng C.H., Li L., Chiu H.F.K., Lok G.K.I., Lu J.P. (2017). The prevalence of insomnia in the general population in China: A meta-analysis. PLoS ONE.

[9] de Freitas S.L.F., Ivo M.L., Figueiredo M.S., de Souza Gerk M.A., Nunes C.B., de Freitas Monteiro F. (2018). Quality of life in adults with sickle cell disease: An integrative review of the literature. Rev. Bras. Enferm..

[10] Mann-Jiles V., Thompson K., Lester J. (2015). Sleep impairment and insomnia in sickle cell disease: A retrospective chart review of clinical and psychological indicators. J. Am. Assoc. Nurse Pract..

[11] Wallen G.R., Minniti C.P., Krumlauf M., Eckes E., Allen D., Oguhebe A., Seamon C., Darbari D.S., Hildesheim M., Yang L. (2014). Sleep disturbance, depression and pain in adults with sickle cell disease. BMC Psychiatry.

[12] Fisher K., Laikin A.M., Sharp K.M.H., Criddle C.A., Palermo T.M., Karlson C.W. (2018). Temporal relationship between daily pain and actigraphy sleep patterns in pediatric sickle cell disease. J. Behav. Med..

[13] Brooks L.J., Koziol S.M., Chiarucci K.M., Berman B.W. (1996). Does Sleep-Disordered Breathing Contribute to the Clinical Severity of Sickle Cell Anemia?. J. Pediatr. Hematol. Oncol..

[14] Sidman J.D., Fry T.L. (1988). Exacerbation of sickle cell disease by obstructive sleep apnea. Arch. Otolaryngol.—Head Neck Surg..

[15] Wittig R.M., Roth T., Keenum A.J., Sarnaik S. (1988). Snoring, Daytime Sleepiness, and Sickle Cell Anemia. Am. J. Dis. Child..

[16] Brennan L.C., Kirkham F.J., Gavlak J.C. (2020). Sleep-disordered breathing and comorbidities: Role of the upper airway and craniofacial skeleton. Nat. Sci. Sleep.

[17] Samuels M.P., Stebbens V.A., Davies S.C., Picton-Jones E., Southall D.P. (1992). Sleep related upper airway obstruction and hypoxaemia in sickle cell disease. Arch. Dis. Child..

[18] Sharma S., Efird J.T., Knupp C., Kadali R., Liles D., Shiue K., Boettger P., Quan S.F. (2015). Sleep disorders in adult sickle cell patients. J. Clin. Sleep Med..

[19] Rogers V.E., Lewin D.S., Winnie G.B., Geiger-Brown J. (2010). Polysomnographic characteristics of a referred sample of children with sickle cell disease. J. Clin. Sleep Med..

[20] Rosen C.L., Debaun M.R., Strunk R.C., Redline S., Seicean S., Craven D.I., Gavlak J.C., Wilkey O., Inusa B., Roberts I. (2014). Obstructive sleep apnea and sickle cell anemia. Pediatrics.

[21] Taherifard E., Taherifard E., Hosseini-Bensenjan M., Sayadi M., Haghpanah S. (2023). The Prevalence of Obstructive Sleep Apnea and Associated Symptoms among Patients with Sickle Cell Disease: A Systematic Review and Meta-analysis. Hemoglobin.

[22] Lee W., Lee S.A., Ryu H.U., Chung Y.S., Kim W.S. (2016). Quality of life in patients with obstructive sleep apnea: Relationship with daytime sleepiness, sleep quality, depression, and apnea severity. Chronic Respir. Dis..

[23] Beninati W., Harris C.D., Herold D.L., Shepard J.W. (1999). The Effect of Snoring and Obstructive Sleep Apnea on the Sleep Quality of Bed Partners. Mayo Clin. Proc..

[24] Balakrishnan G., Burli D., Behbehani K., Burk J.R., Lucas E.A. Comparison of a Sleep Quality Index between Normal and Obstructive Sleep Apnea Patients. Proceedings of the 2005 IEEE Engineering in Medicine and Biology 27th Annual Conference.

[25] Phillips B.G., Kato M., Narkiewicz K., Choe I., Somers V.K. (2000). Increases in leptin levels, sympathetic drive, and weight gain in obstructive sleep apnea. Am. J. Physiol. Heart Circ. Physiol..

[26] Olaithe M., Bucks R.S. (2013). Executive Dysfunction in OSA Before and After Treatment: A Meta-Analysis. Sleep.

[27] Olaithe M., Bucks R.S., Hillman D.R., Eastwood P.R. (2018). Cognitive deficits in obstructive sleep apnea: Insights from a meta-review and comparison with deficits observed in COPD, insomnia, and sleep deprivation. Sleep Med. Rev..

[28] Bucks R.S., Olaithe M., Eastwood P. (2013). Neurocognitive function in obstructive sleep apnoea: A meta-review. Respirology.

[29] Slater G., Steier J. (2012). Excessive daytime sleepiness in sleep disorders. J. Thorac. Dis..

[30] Vicente E., Marin J.M., Carrizo S.J., Osuna C.S., González R., Marin-Oto M., Forner M., Vicente P., Cubero P., Gil A.V. (2016). Upper airway and systemic inflammation in obstructive sleep apnoea. Eur. Respir. J..

[31] Bouloukaki I., Mermigkis C., Tzanakis N., Kallergis E., Moniaki V., Mauroudi E., Schiza S.E. (2017). Evaluation of Inflammatory Markers in a Large Sample of Obstructive Sleep Apnea Patients without Comorbidities. Mediators Inflamm..

[32] Lavie L. (2015). Oxidative stress in obstructive sleep apnea and intermittent hypoxia e Revisited e The bad ugly and good: Implications to the heart and brain. Sleep Med. Rev..

[33] Passali D., Corallo G., Yaremchuk S., Longini M., Proietti F., Passali G.C., Bellussi L. (2015). Stress ossidativo nei pazienti con diagnosi di sindrome delle apnee ostruttive notturne. Acta Otorhinolaryngol. Ital..

[34] Kirkham F.J., Hewes D.K.M., Prengler M., Wade A., Lane R., Evans J.P.M. (2001). Nocturnal hypoxaemia and central-nervous-system events in sickle-cell disease. Lancet.

[35] Okoli K., Irani F., Horvath W. (2009). Pathophysiologic considerations for the interactions between obstructive sleep apnea and sickle hemoglobinopathies. Med. Hypotheses.

[36] Kaleyias J., Mostofi N., Grant M., Coleman C., Luck L., Dampier C., Kothare S.V. (2008). Severity of obstructive sleep apnea in children with sickle cell disease. J. Pediatr. Hematol. Oncol..

[37] Hargrave D.R., Wade A., Evans J.P.M., Hewes D.K.M., Kirkham F.J. (2003). Nocturnal oxygen saturation and painful sickle cell crises in children. Blood.

[38] Kepplinger J., Barlinn K., Boehme A.K., Gerber J., Puetz V., Pallesen L., Schrempf W., Dzialowski I., Albright K.C., Alexandrov A.V. (2014). Association of sleep apnea with clinically silent microvascular brain tissue changes in acute cerebral ischemia. J. Neurol..

[39] Durgan D.J., Bryan R.M. (2012). Cerebrovascular Consequences of Obstructive Sleep Apnea. J. Am. Heart Assoc..

[40] Alvarez-Sabín J., Romero O., Delgado P., Quintana M., Santamarina E., Ferré A., Maisterra O., Riba-Llena I., Montaner J., Sampol G. (2018). Obstructive sleep apnea and silent cerebral infarction in hypertensive individuals. J. Sleep Res..

[41] Fabbri M., Beracci A., Martoni M., Meneo D., Tonetti L., Natale V. (2021). Measuring subjective sleep quality: A review. Int. J. Environ. Res. Public Health.

[42] Harrison E.I., Roth R.H., Lobo J.M., Kang H., Logan J., Patel S.R., Kapur V.K., Kwon Y. (2021). Sleep time and efficiency in patients undergoing laboratory-based polysomnography. J. Clin. Sleep Med..

[43] Liguori C., Mombelli S., Fernandes M., Zucconi M., Plazzi G., Ferini-Strambi L., Logroscino G., Mercuri N.B., Filardi M. (2023). The evolving role of quantitative actigraphy in clinical sleep medicine. Sleep Med. Rev..

[44] Chervin R.D., Hedger K., Dillon J.E., Pituch K.J. (2000). Pediatric sleep questionnaire (PSQ): Validity and reliability of scales for sleep-disordered breathing, snoring, sleepiness, and behavioral problems. Sleep Med..

[45] Incerti Parenti S., Fiordelli A., Bartolucci M.L., Martina S., D’Antò V., Alessandri-Bonetti G. (2021). Diagnostic accuracy of screening questionnaires for obstructive sleep apnea in children: A systematic review and meta-analysis. Sleep Med. Rev..

[46] Johns M.W. (1991). A new method for measuring daytime sleepiness: The Epworth sleepiness scale. Sleep.

[47] Owens J., Spirito A., McGuinn M. (2000). The Children’s Sleep Habits Questionnaire (CSHQ): Psychometric Properties of A Survey Instrument for School-Aged Children. Sleep.

[48] Bonuck K.A., Goodlin-Jones B.L., Schechter C., Owens J. (2017). Modified Children’s sleep habits questionnaire for behavioral sleep problems: A validation study. Sleep Health.

[49] Natale V., Léger D., Bayon V., Erbacci A., Tonetti L., Fabbri M., Martoni M. (2015). The consensus sleep diary: Quantitative criteria for primary insomnia diagnosis. Psychosom. Med..

[50] Markun L.C., Sampat A. (2020). Clinician-Focused Overview and Developments in Polysomnography. Curr. Sleep Med. Rep..

[51] Moss D., Urschitz M.S., Von Bodman A., Eitner S., Noehren A., Urschitz-Duprat P.M., Schlaud M., Poets C.F. (2005). Reference values for nocturnal home polysomnography in primary schoolchildren. Pediatr. Res..

[52] Berry R.B., Brooks R., Gamaldo C.E., Harding S.M., Marcus C.L., Vaughn B.V. (2013). The AASM Manual for the Scoring of Sleep and Associated Events. Am. Acad. Sleep Med..

[53] Levendowski D.J., Zack N., Rao S., Wong K., Gendreau M., Kranzler J., Zavora T., Westbrook P.R. (2009). Assessment of the test-retest reliability of laboratory polysomnography. Sleep Breath..

[54] Russo K., Greenhill J., Burgess S. (2021). Home (Level 2) polysomnography is feasible in children with suspected sleep disorders. Sleep Med..

[55] Acker J.G., Becker-Carus C., Büttner-Teleaga A., Cassel W., Danker-Hopfe H., Dück A., Frohn C., Hein H., Penzel T., Rodenbeck A. (2021). The role of actigraphy in sleep medicine. Somnologie.

[56] Patterson M.R., Nunes A.A.S., Gerstel D., Pilkar R., Guthrie T., Neishabouri A., Guo C.C. (2023). 40 years of actigraphy in sleep medicine and current state of the art algorithms. NPJ Digit. Med..

[57] Yavuz-Kodat E., Reynaud E., Geoffray M.-M., Limousin N., Franco P., Bourgin P., Schroder C.M. (2019). Validity of Actigraphy Compared to Polysomnography for Sleep Assessment in Children With Autism Spectrum Disorder. Front. Psychiatry.

[58] Lin C.H., Chen C.H., Hong S.Y., Chou I.C., Liang S.J., Hang L.W. (2021). Polysomnography is an important method for diagnosing pediatric sleep problems: Experience of one children’s hospital. Children.

[59] Moher D., Liberati A., Tetzlaff J., Altman D.G., Grp P. (2009). Preferred Reporting Items for Systematic Reviews and Meta-Analyses: The PRISMA Statement (Reprinted from Annals of Internal Medicine). Phys. Ther..

[60] Hirshkowitz M., Whiton K., Albert S.M., Alessi C., Bruni O., DonCarlos L., Hazen N., Herman J., Katz E.S., Kheirandish-Gozal L. (2015). National sleep foundation’s sleep time duration recommendations: Methodology and results summary. Sleep Health.

[61] Ohayon M., Wickwire E.M., Hirshkowitz M., Albert S.M., Avidan A., Daly F.J., Dauvilliers Y., Ferri R., Fung C., Gozal D. (2017). National Sleep Foundation’s sleep quality recommendations: First report. Sleep Health J. Natl. Sleep Found..

[62] Paruthi S., Brooks L.J., D’Ambrosio C., Hall W.A., Kotagal S., Lloyd R.M., Malow B.A., Maski K., Nichols C., Quan S.F. (2016). Recommended amount of sleep for pediatric populations: A consensus statement of the American Academy of Sleep Medicine. J. Clin. Sleep Med..

[63] Watson N.F., Badr M.S., Belenky G., Bliwise D.L., Buxton O.M., Buysse D., Dinges D.F., Gangwisch J., Grandner M.A., Kushida C. (2015). Recommended amount of sleep for a healthy adult: A joint consensus statement of the American Academy of Sleep Medicine and Sleep Research Society. J. Clin. Sleep Med..

[64] Njamnshi A.K., Ngarka L., Seke P.F.E., Njoh A.A., Mbong E.N., Chokote E.T., Fonsah J.Y., Koki N., Muna W.F.T. (2013). Actigraphy in the assessment of sleep patterns in sickle cell disease patients in Cameroon (Sub-Saharan Africa). J. Neurol. Sci..

[65] Biggs S.N., Lushington K., James Martin A., van den Heuvel C., Declan Kennedy J. (2013). Gender, socioeconomic, and ethnic differences in sleep patterns in school-aged children. Sleep Med..

[66] Ohayon M., Carskadon M., Guilleminault C., Vitiello M. (2004). Meta-Analysis of Quantitative Sleep Parameters From Childhood to Old Age in Healthy Individuals: Developing Normative Sleep Values Across the Human Lifespan. Sleep.

[67] Johnson D., Jackson C.L., Williams N.J., Alcántara C. (2019). Are sleep patterns influenced by race/ethnicity—A marker of relative advantage or disadvantage? Evidence to date. Nat. Sci. Sleep.

[68] Valrie C.R., Gil K.M., Redding-Lallinger R., Daeschner C. (2007). Brief report: Sleep in children with sickle cell disease: An analysis of daily diaries utilizing multilevel models. J. Pediatr. Psychol..

[69] Blader C.J., Koplewicz S.H., Abikoff H., Foley C. (1997). Sleep Problems of Elementary School Children. Arch. Pediatr. Adolesc. Med..

[70] Nelson T.D., Lundahl A., Molfese D.L., Waford R.N., Roman A., Gozal D., Molfese V.J., Ferguson M.C. (2014). Estimating child sleep from parent report of time in bed: Development and evaluation of adjustment approaches. J. Pediatr. Psychol..

[71] Combs D., Goodwin J.L., Quan S.F., Morgan W.J., Hsu C.H., Edgin J.O., Parthasarathy S. (2019). Mother knows best? Comparing child report and parent report of sleep parameters with polysomnography. J. Clin. Sleep Med..

[72] Yuan H., Hill E.A., Kyle S.D., Doherty A. (2024). A systematic review of the performance of actigraphy in measuring sleep stages. J. Sleep Res..

[73] Murata E., Kato-Nishimura K., Taniike M., Mohri I. (2020). Evaluation of the validity of psychological preparation for children undergoing polysomnography. J. Clin. Sleep Med..

[74] Daniel L.C., Grant M., Kothare S.V., Dampier C., Barakat L.P. (2010). Sleep patterns in pediatric sickle cell disease. Pediatr. Blood Cancer.

[75] Kölbel M., Kirkham F.J., Iles R.K., Stotesbury H., Halstead E., Brenchley C., Sahota S., Dimitriou D. (2022). Exploring the relationship of sleep, cognition, and cortisol in sickle cell disease. Compr. Psychoneuroendocrinol..

[76] Mascarenhas M.I., Loureiro H.C., Ferreira T., Dias A. (2015). Sleep pathology characterization in sickle cell disease: Case-control study. Pediatr. Pulmonol..

[77] Valrie C.R., Trout K.L., Bond K.E., Huber N.L., Alston K.J., Sufrinko A.M., Everhart E., Fuh B.R. (2018). Sleep Problem Risk for Adolescents with Sickle Cell Disease: Sociodemographic, Physical, and Disease-related Correlates. J. Pediatr. Hematol. Oncol..

[78] Strauss T., Sin S., Marcus C.L., Mason T.B.A., McDonough J.M., Allen J.L., Caboot J.B., Bowdre C.Y., Jawad A.F., Smith-Whitley K. (2012). Upper airway lymphoid tissue size in children with sickle cell disease. Chest.

