# Supplementary material for: Sleep Behaviour in Sickle Cell Disease: A Systematic Review and Meta-Analysis
Source: Children (Basel). 2024 Dec 26;12(1):21. doi: 10.3390/children12010021 (PMC11763464; doi:10.3390/children12010021)

Supplementary Material Table S1: Quality assessment table.

AUTHOR	Q1	Q2	Q3	Q4	Q5	Q6	Q7	Q8	Q9	Q10	Q11	Q12	GRADE	SCORE
VALRIE 2006	1	0	1	1	1	1	1	0	1	1	1	0	9	Low
SOUZA & VIEGAS 2007	0	1	1	0	0	0	1	0	1	1	1	1	7	Low
VALRIE 2007	1	1	0	0	1	0	1	1	1	1	1	1	9	Low
KALEYIAS 2008	1	1	1	1	0	1	1	1	1	0	1	1	10	Moderate
FERREIRA 2009	1	1	1	1	0	1	1	0	1	0	1	0	8	Low
ROGERS 2009	1	1	1	1	1	1	1	1	1	1	1	1	12	High
DANIEL 2010	1	1	1	1	1	1	1	1	1	1	1	1	12	High
MARTINS 2010	1	0	1	1	0	0	1	0	1	0	1	0	6	Low
ROGERS 2010	1	1	1	1	1	1	1	1	1	1	1	1	12	High
ROGERS 2011	1	1	1	1	1	0	1	1	1	1	1	1	11	Moderate
MULLIN 2012	1	1	1	1	1	1	1	1	1	1	1	1	12	High
STRAUSS 2012	1	1	1	1	0	0	1	1	1	1	1	1	10	Moderate
FINCH 2013	1	0	1	1	0	1	1	0	1	0	1	0	7	Low
NJAMNSHI 2013	1	0	0	1	0	0	1	0	1	1	1	0	6	Low
KATZ 2014	1	1	1	1	1	1	1	1	1	1	1	1	12	High
ROSEN 2014	1	1	1	1	1	1	1	1	1	1	1	1	12	High
LOUREIRO 2015	1	0	1	1	1	1	1	0	1	1	1	0	9	Low
MASCARENHAS 2015	1	1	1	1	1	1	1	1	1	1	1	1	12	High
MOSCOU-JACKSON 2015	1	1	0	1	1	1	1	1	1	1	1	1	11	Moderate
NARANG 2015	1	1	1	1	1	1	1	1	1	1	1	1	12	High
AL-OTAIBI 2017	1	0	1	1	0	1	1	1	1	0	1	0	8	Low
DOWNES 2017	1	1	1	1	0	1	1	0	1	1	1	0	9	Low
AL-OTAIBI 2018	1	0	1	1	0	1	1	0	1	0	1	0	7	Low
FISHER 2018	1	0	1	1	1	1	1	0	1	0	1	0	8	Low
KATZ 2018	1	1	1	1	1	1	1	0	1	1	1	1	11	Moderate
VALRIE 2018	1	0	0	0	1	1	1	0	1	1	1	1	8	Low
VALRIE 2019	1	1	1	1	1	1	1	1	1	1	1	1	12	High
VALRIE 2020	1	1	1	1	1	1	1	0	1	1	1	1	11	Moderate
KÖLBEL 2022	1	1	1	1	0	1	1	0	1	1	1	1	10	Moderate

Supplementary Material Figure S1: Forest Plot for total sleep time measured by polysomnography.

Author	Year	Hedges'*g*	Std. Error	*CI 95%*	*p*-value	Weight (%)
Strauss	2012	-0.39	0.24	-0.85,0.08	0.10	7.68 (44.5%)
Mascarenhas	2015	0.10	0.18	-0.25,0.44	0.58	6.26 (55.5%)
**Overall**		**-0.12**	**0.24**	**-0.59,0.35**	**0.62**	

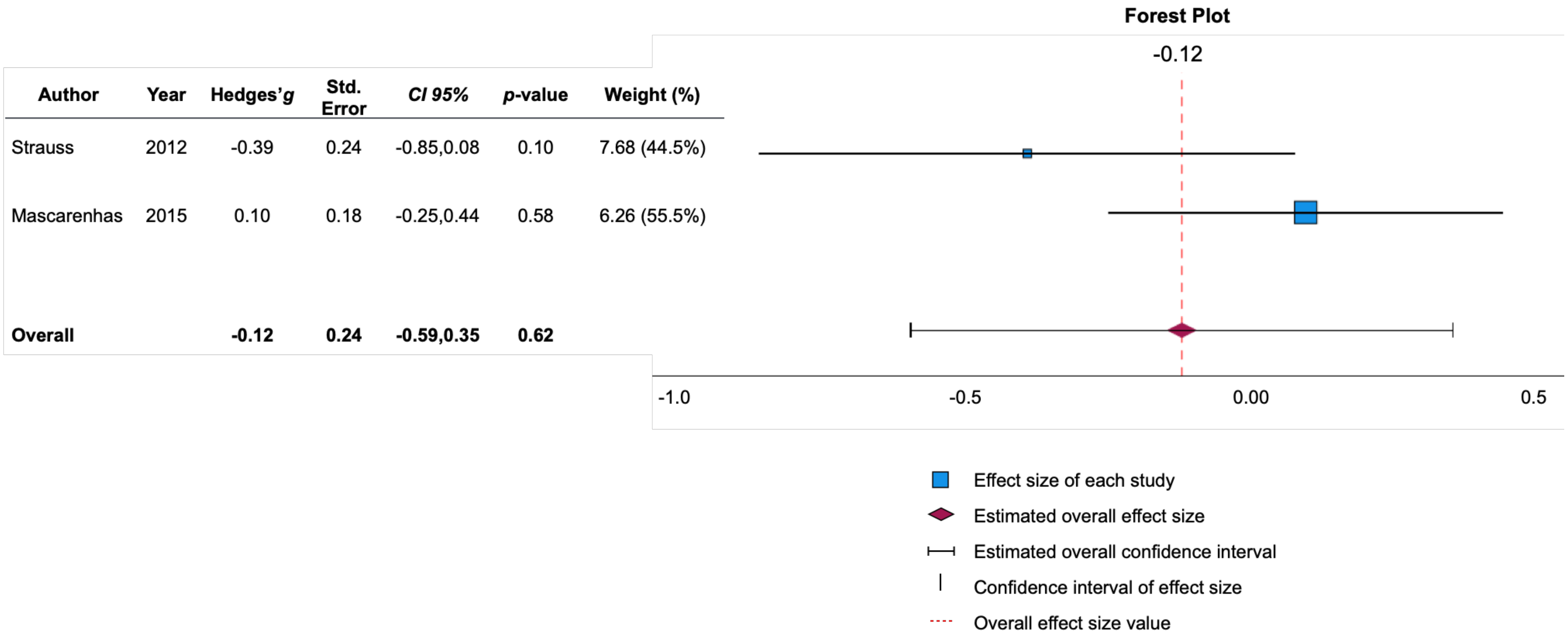

Supplementary Material Figure S2: Forest Plot for total sleep time measured by actigraphy.

Author	Year	Hedges'*g*	Std. Error	*CI 95%*	*p*-value	Weight (%)
Njamnshi	2013	-3.54	0.66	-4.84,-2.24	< 0.001	0.13 (48.8%)
Kölbel	2022	0.27	0.31	-0.33,0.88	0.38	0.14 (51.2%)
**Overall**		**-1.59**	**1.90**	**-5.32,2.14**	**0.40**	

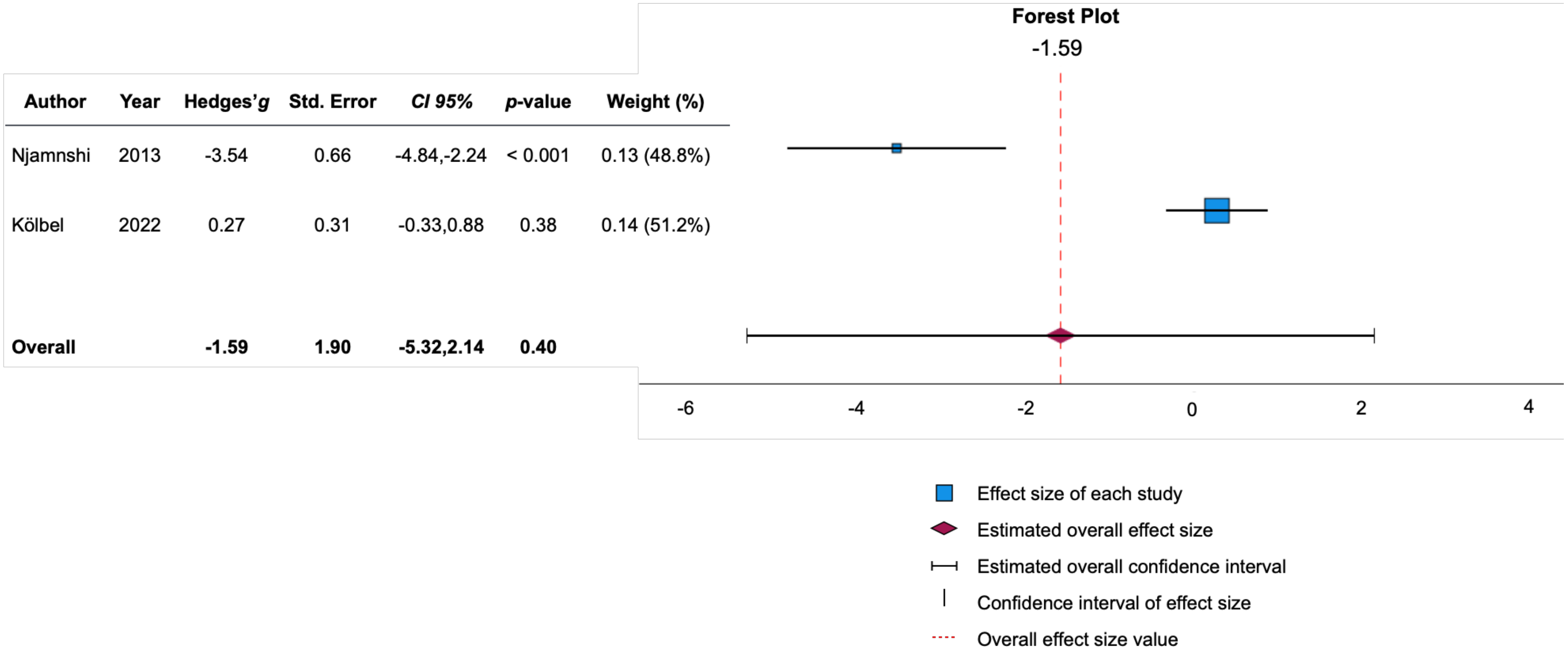

Supplementary Material Figure S3: Forest Plot for total sleep time measured by sleep diary.

Author	Year	Hedges'*g*	Std. Error	*CI 95%*	*p*-value	Weight (%)
Daniel	2010	0.08	0.19	-0.30,0.46	0.69	5.84 (48.2%)
Valerie	2018	0.65	0.16	0.34,0.97	<0.001	6.26 (51.8%)
**Overall**		**0.38**	**0.29**	**-0.19,0.94**	**0.19**	

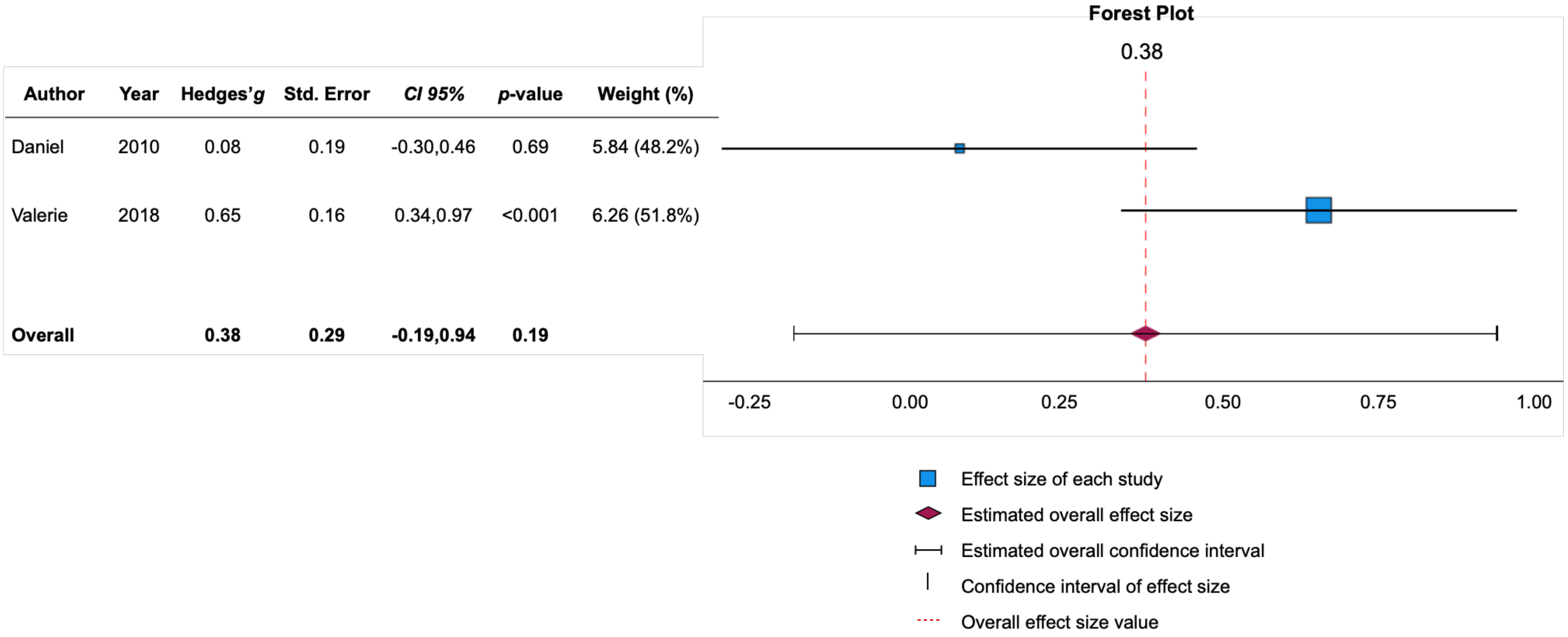

Supplementary Material Figure S4: Forest Plot for sleep onset latency measured by actigraphy.

Author	Year	Hedges'*g*	Std. Error	*CI 95%*	*p*-value	Weight (%)
Njamnshi	2013	-2.72	0.57	-3.83,-1.61	< 0.001	0.12 (48.8%)
Kölbel	2022	0.35	0.31	-0.26,0.95	0.26	0.22 (51.2%)
**Overall**		**-1.15**	**1.53**	**-4.15,1.86**	**0.45**	

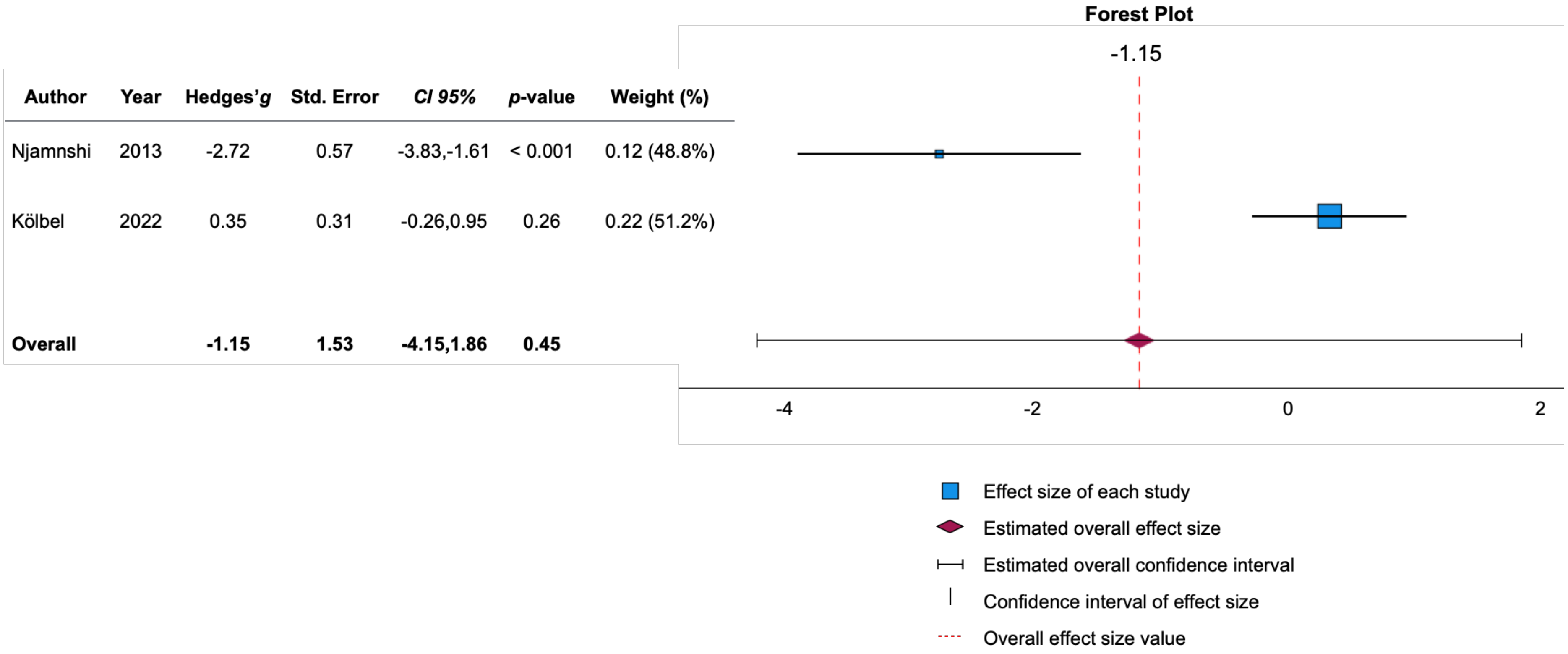

Supplement: Supplementary file 1 [file children-12-00021-s001.zip › children-3308976-supplementary.pdf]
